# Conceptual framework for the insect metamorphosis from larvae to pupae by transcriptomic profiling, a case study of *Helicoverpa armigera* (Lepidoptera: Noctuidae)

**DOI:** 10.1186/s12864-022-08807-y

**Published:** 2022-08-13

**Authors:** Xinxin Gao, Jihong Zhang, Peipei Wu, Ruihao Shu, Huan Zhang, Qilian Qin, Qian Meng

**Affiliations:** 1 State Key Laboratory of Integrated Management of Pest Insects and Rodents, Institute of Zoology, Chinese Academy of Sciences, Beijing, China; 2 University of Chinese Academy of Sciences, Beijing, China; 3 Institute of Edible Fungi, Shanghai Academy of Agricultural Sciences, Shanghai, China

**Keywords:** *Helicoverpa armigera*, Metamorphosis, Transcriptomic analysis

## Abstract

**Background:**

Insect metamorphosis from larvae to pupae is one of the most important stages of insect life history. Relatively comprehensive information related to gene transcription profiles during lepidopteran metamorphosis is required to understand the molecular mechanism underlying this important stage. We conducted transcriptional profiling of the brain and fat body of the cotton bollworm *Helicoverpa armigera* (Lepidoptera: Noctuidae) during its transition from last instar larva into pupa to explore the physiological processes associated with different phases of metamorphosis.

**Results:**

During metamorphosis, the differences in gene expression patterns and the number of differentially expressed genes in the fat body were found to be greater than those in the brain. Each stage had a specific gene expression pattern, which contributed to different physiological changes. A decrease in juvenile hormone levels at the feeding stage is associated with increased expression levels of two genes (juvenile hormone esterase, juvenile hormone epoxide hydrolase). The expression levels of neuropeptides were highly expressed at the feeding stage and the initiation of the wandering stage and less expressed at the prepupal stage and the initiation of the pupal stage. The transcription levels of many hormone (or neuropeptide) receptors were specifically increased at the initiation of the wandering stage in comparison with other stages. The expression levels of many autophagy-related genes in the fat body were found to be gradually upregulated during metamorphosis. The activation of apoptosis was probably related to enhanced expression of many key genes (Apaf1, IAP-binding motif 1 like, cathepsins, caspases). Active proliferation might be associated with enhanced expression levels in several factors (JNK pathway: jun-D; TGF-β pathway: decapentaplegic, glass bottom boat; insulin pathway: insulin-like peptides from the fat body; Wnt pathway: wntless, TCF/Pangolin).

**Conclusions:**

This study revealed several vital physiological processes and molecular events of metamorphosis and provided valuable information for illustrating the process of insect metamorphosis from larvae to pupae.

**Supplementary Information:**

The online version contains supplementary material available at 10.1186/s12864-022-08807-y.

## Background

Insect metamorphosis from larvae to pupae occurs when insects reach a certain critical weight [1] and is regulated by the cooperation of juvenile hormone (JH) and ecdysone (E). The level of JH in the last instar larva of Lepidoptera greatly decreases to a low level before the wandering stage, remains at a low level at the initiation of the wandering stage, rises to a high level during the prepupal stage and decreases to a low level at the initiation of the pupal stage [2]. The level of E increases at the initiation of the wandering stage, rises to a high level during the prepupal stage and decreases to a low level at the initiation of the pupal stage [2]. As the titer of E rises, the old tissues of insects are separated and degraded, and new tissues are formed [3].

JH is produced by corpora allata (CA). JH acid methyltransferase (JHAMT) is the key rate-limiting enzyme controlling the titer of JH in the last instar of insects. Reduction in *JHAMT* transcription results in a decrease in the JH titer [4]. In the cricket, the TGF-β signaling ligands decapentaplegic (Dpp) and myoglianin regulate the transcription of *JHAMT*. Specifically, Dpp signaling can enhance *JHAMT* transcription, while myoglianin signaling can reduce *JHAMT* transcription by inhibiting Dpp signaling [5, 6]. Myoglianin inhibition of *JHAMT* transcription has been identified in crickets, cockroaches and *Tribolium castaneum* [6–8]. In *Drosophila*, myoglianin expression is induced by the transcription factor Max‐binding protein (MNT) [9]. It is unclear whether the regulatory role of MNT on myoglianin is also present in other insects. Juvenile hormone binding protein (JHBP) delivers JH to cells. RNA interference of JHBP causes a decrease in the JH titer in *H. armigera* [10, 11]. JH degradation is regulated by juvenile hormone esterase (JHE) and juvenile hormone epoxide hydrolase (JHEH) [12, 13].

After JH decreases to a certain titer, the prothoracic gland (PG) begins to secrete E under the stimulation of prothoracicotropic hormone (PTTH). A small amount of E triggers biosynthesis and secretion of a large amount of E [14]. 20-hydroxyecdysone (20E), an active metabolite of E, triggers the beginning of insect metamorphosis. Interestingly, ecdysone production is also mediated by TGF-β signaling; the TGF-β ligand Dpp inhibits ecdysone production, while activin signaling promotes ecdysone production [15–17].

The insulin-signaling pathway is an important pathway in insect development and metamorphosis, which interacts with JH and E. On the one hand, insulin signaling can promote the biosynthesis of E in the PG by directly promoting the expression of ecdysone biosynthetic genes and inhibiting FOXO activity [18–20]. On the other hand, ecdysone signaling can antagonize insulin signaling by decreasing phosphorylation levels of insulin receptor and serine-threonine protein kinase Akt [21]. In addition, E and JH form negative feedback regulation by inhibiting each other's biosynthesis [22, 23].

Autophagy, apoptosis and cell proliferation of larval tissues regulated by E are vital components of metamorphosis. Autophagy-related genes (ATGs) play vital roles in the autophagy process. Autophagy-related proteins are involved in four steps of the autophagy process, including induction, cargo recognition and selectivity, autophagosome formation, vesicle fusion and autophagosome breakdown [24]. In addition, autophagy precedes and mediates 20E-induced apoptosis [25, 26]. 20E triggers autophagy and apoptosis by changing the intracellular concentration of Ca^2+^ [25]. Cytochrome c released from mitochondria promotes caspase-9 and caspase-3 activation to induce apoptosis in vertebrates, but it has little effect on inducing apoptosis in *C. elegans* and *Drosophila* [27–30]. However, cytochrome c release is probably required during apoptosis in lepidopteran insects [26]. Caspases are expressed as procaspases, and the activation of caspases triggers apoptosis by degrading critical cellular substrates [31]. Caspase DRONC (the initiation caspase) and caspase 1 (the effector caspase) play vital roles in apoptosis in *Drosophila* and lepidopteran insects, respectively [32, 33]. 20E induces autophagy and caspase activation during metamorphosis [34].

The brain is an important organ that controls insect hormone secretion and behavior. The main functions of fat bodies include energy metabolism, nutrient storage, detoxification and the immune response [35]. Neuropeptides are extremely diverse and play an important role in many physiological processes. In addition, they often cooperate with each other to regulate certain behaviors and have multiple functions [36]. The neuropeptide SIFamide is involved in translation of hunger signals [37]. Dopamine and dopamine receptors are related to the regulation of motor behavior, food-seeking behavior, and the initiation of metamorphosis [38–40]. 5-Hydroxytryptamine and its receptor are involved in the modulation of feeding and gut contractions, regulation of larval locomotion [41, 42]. Octopamine is related to aggression, appetitive and aversive memory recall, and modulation of behavior and metabolism [43–45]. The changes in gene transcription profiles in the brain of lepidopteran insects, especially the changes in neuropeptides, during metamorphosis from larva to pupa are unknown.

*Helicoverpa armigera* is a serious agricultural pest worldwide. Rapid evolution of resistance to chemical pesticides and *Bacillus thuringiensis toxin* was observed in this pest. In recent years, great progress has been made in hormonal regulation of insect development using *H. armigera* as a model [46]. Understanding of the physiological processes and molecular mechanisms of development of cotton bollworm will help to find more effective control methods. Although the molecular regulatory mechanisms of hormone (JH and 20E) regulation and autophagy of insect metamorphosis have been studied, the specific physiological processes of *H. armigera* from larvae to pupae are not clear. Little is known about the molecular mechanism of cell proliferation in lepidopteran insects during metamorphosis from larvae to pupae. Comparative transcriptome analysis was used to analyze specific gene expression profiles in the brain and fat body of *H. armigera* at different stages of metamorphosis in this study. In addition, we further studied several important physiological processes of metamorphosis, including regulation of JH and ecdysone signaling pathways, autophagy, apoptosis and cell proliferation. Our primary aim was to assess the changes in crucial genes and signaling pathways during different physiological processes from larvae to pupae to provide more references for studies of insect metamorphosis.

## Results

### Overall transcriptome analysis of the brain and fat body of *H. armigera* at different physiological stages

**Fig. 1:**
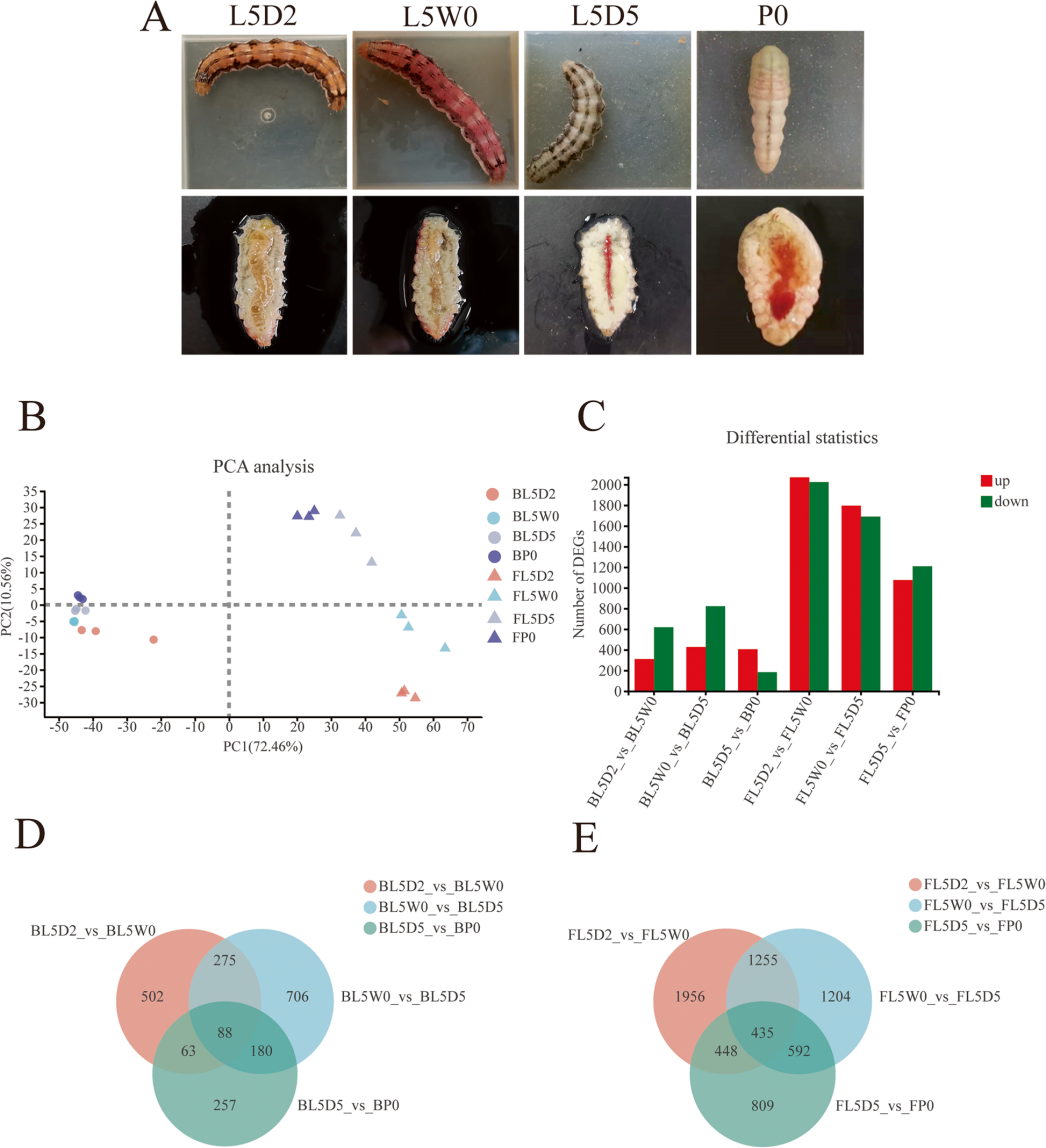
Phenotypes and transcriptomic overview of *Helicoverpa armigera* at different metamorphosis stages. **A** Metamorphosis phenotypes of the last instar of *H. armigera* larvae at different stages. The pictures below were dissected insects revealing their midgut and fat bodies. **B** PCA plot of global gene expression of all samples. **C** Total number of DEGs at different stages. **D** Venn diagram exhibiting common and unique DEGs in the brain at different stages. **E** Venn diagram exhibiting common and unique DEGs in the fat body at different stages. L5D2: the feeding stage; L5W0: the initiation of the wandering stage; L5D5: the prepupal stage; P0: the initiation of the pupal stage

The metamorphosis of *H. armigera* from larvae to pupae underwent several stages. From the first day to the third day of the fifth instar, larvae fed continuously and accumulated energy to prepare for metamorphosis. On the third day of the fifth instar, the beginning of metamorphosis, larvae stopped feeding and started to wander. From the third day to the sixth day, the fifth instar larvae entered the prepupal stage, and the larval tissues underwent remodeling. From about the sixth day of the fifth instar, larvae gradually completed the transition from larvae to pupae. In this study, four key stages were chosen to study metamorphosis from larvae to pupae, including the feeding stage (L5D2: the second day of the fifth instar larvae), the initiation of the wandering stage (L5W0: the third day of the fifth instar larvae), the prepupal stage (L5D5: the fifth day of fifth instar larvae), and the initiation of the pupal stage (P0) (Fig. 1A).

Transcriptome analysis was conducted to investigate the changes in gene expression in the brain and fat body of *H. armigera* at four different physiological stages, including the feeding stage (L5D2) (the brain: BL5D2; the fat body: FL5D2), the initiation of the wandering stage (L5W0) (the brain: BL5W0; the fat body: FL5W0), the prepupal stage (L5D5) (the brain: BL5D5; the fat body: FL5D5), and the initiation of the pupal stage (P0) (the brain: BP0; the fat body: FP0). A total of 174.67 Gb clean data of 24 samples was obtained, and the amount of clean data for each example exceeded 6.34 Gb with the percentage of Q30 ≥ 94.64 (Additional file 1: Table S1). For each sample, 68.8-81.3% of the clean reads were mapped to the *H. armigera* genome (Additional file 2: Table S2).

The different stages of metamorphosis from larva to pupa were separated by a PCA map (Fig. 1B). PCA showed that different stages of metamorphosis were significantly different. The samples from the brain at different physiological stages were clustered more closely than samples from the fat body, which indicated that the changes in gene expression in the fat body were greater than those in the brain.

|log_2_ FC |≥ 1 and FDR adjusted *P value* < 0.05 were set as thresholds, and differentially expressed genes (DEGs) were obtained from the two different tissues at four different physiological stages (Additional file 3: Table S3). A total of 928 DEGs were identified in BL5D2 vs BL5W0 (310 upregulated and 618 downregulated). A total of 1249 DEGs were identified in BL5W0 vs BL5D5 (427 upregulated and 822 downregulated). A total of 588 DEGs were identified in BL5D5 vs BP0 (405 upregulated and 183 downregulated). A total of 4094 DEGs were identified in FL5D2 vs FL5W0 (2070 upregulated and 2024 downregulated). A total of 3486 DEGs were identified in FL5W0 vs FL5D5 (1796 upregulated and 1690 downregulated). A total of 2284 DEGs were identified in FL5D5 vs FP0 (1075 upregulated and 1209 downregulated). The numbers of DEGs in the brain at different physiological stages were lower than those in the fat body (Fig. 1C). Among the comparisons of different stages, 88 and 435 shared DEGs were found in the brain and fat body, respectively (Fig. 1D and E).

### GO analysis of DEGs

**Fig. 2:**
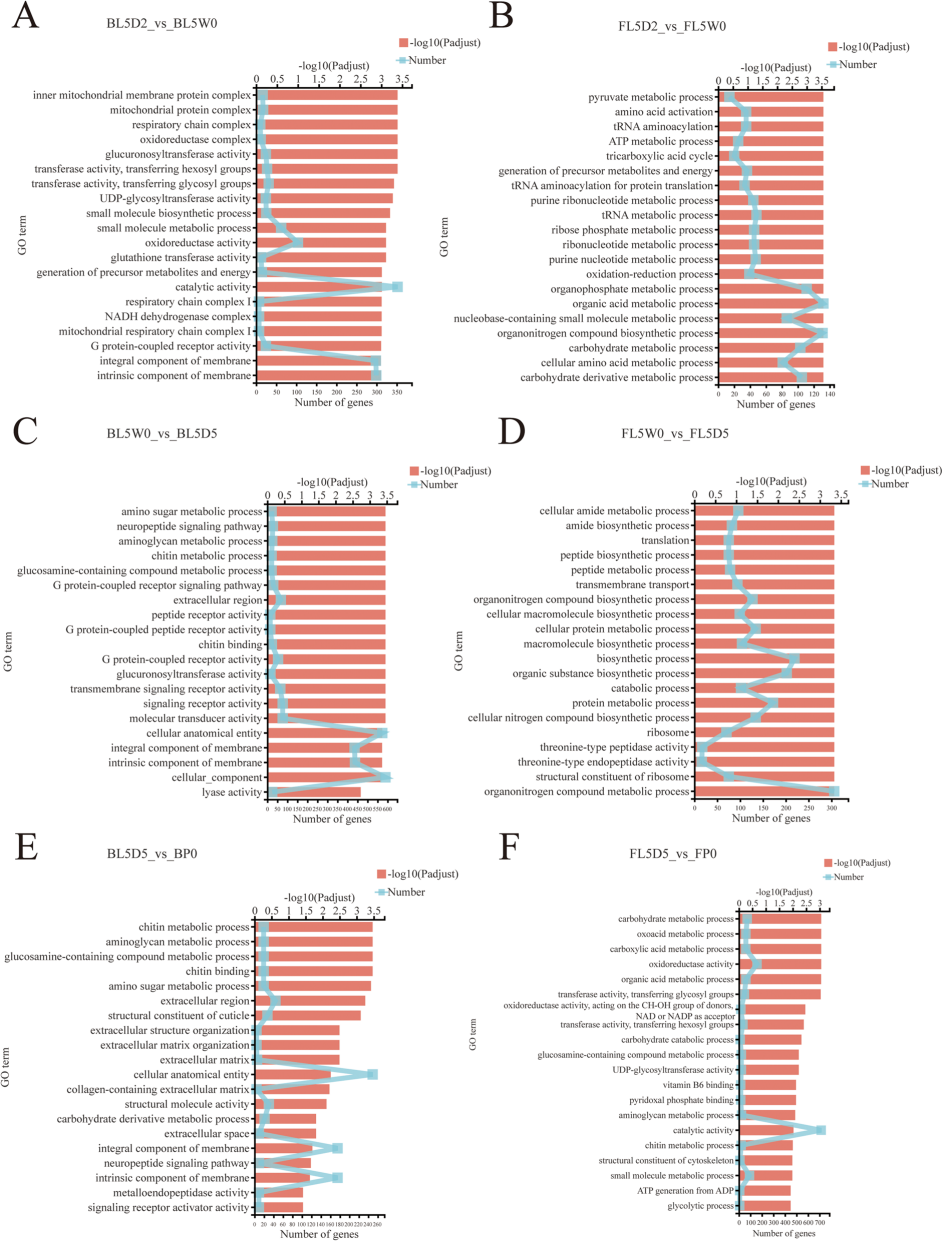
GO enrichment analysis of DEGs of *Helicoverpa armigera* at different stages. **A** Enriched GO analysis of DEGs between BL5D2 and BL5W0. **B** Enriched GO analysis of DEGs between FL5D2 and FL5W0. **C** Enriched GO analysis of DEGs between BL5W0 and BL5D5. **D** Enriched GO analysis of DEGs between FL5W0 and FL5D5. **E** Enriched GO analysis of DEGs between BL5D5 and BP0. **F** Enriched GO analysis of DEGs between FL5D5 and FP0. BL5D2: the brain at the feeding stage; FL5D2: the fat body at the feeding stage; BL5W0: the brain at the initiation of the wandering stage; FL5W0: the fat body at the initiation of the wandering stage; BL5D5: the brain at the prepupal stage; FL5D5: the fat body at the prepupal stage; BP0: the brain at the initiation of the pupal stage; FP0: the fat body at the initiation of the pupal stage

To investigate the functional categories of the DEGs in the brain and fat body at four different physiological stages (L5D2, L5W0, L5D5 and P0), GO enrichment analysis was carried out. The top 20 GO enrichment categories of DEGs in the brain and fat body at different stages are shown in Fig. 2.

#### Initial phase of metamorphosis

Figures 2A and B show the GO enriched categories of the last instar larvae of *H. armigera* from the feeding stage to the initiation of the wandering stage (L5D2 to L5W0). During the feeding stage, the larvae fed continuously, and the fat body content increased (Fig. 1A: L5D2). At the initiation of the wandering stage, the larvae stopped feeding, emptied midgut and began to wander (Fig. 1A: L5W0). GO enrichment analysis revealed that the DEGs in BL5D2 vs BL5W0 were enriched in the inner mitochondrial membrane protein complex, mitochondrial protein complex and respiratory chain complex (Fig. 2A). Mitochondria are the site of energy production in cells. The genes associated with the inner mitochondrial membrane protein complex, including NADH dehydrogenase subunits, cytochrome b-c1 complex subunit 9, cytochrome c oxidase subunit 6A1, ATP synthase-coupling factor 6, and ATP synthase subunits, were highly expressed at the feeding stage (L5D2) (Additional file 4: Table S4), indicating that the brain had high energy metabolism at the feeding stage.

The DEGs in FL5D2 vs FL5W0 were enriched in the pyruvate metabolic process, amino acid activation and tRNA aminoacylation (Fig. 2B). Aminoacyl tRNA synthetases are required for protein synthesis by catalyzing the linking of amino acids to their cognate tRNAs [47]. The genes related to amino acid activation (aminoacyl tRNA ligases) were highly expressed during the feeding stage (FL5D2) (Additional file 4: Table S4), implying that protein synthesis was more active during the feeding stage, which was associated with a continuous increase in the fat body content.

#### Metamorphosis progression

Figures 2C and D show the GO enriched categories of the last instar larvae of *H. armigera* from the initiation of the wandering stage to the prepupal stage (L5W0 to L5D5). During the prepupal stage, the larval body size shrank under the stimulation of E. The fat body was separated and degraded, and the midgut turned red (Fig. 1A: L5D5). GO enrichment analysis revealed that the DEGs in BL5W0 vs BL5D5 were enriched in amino sugar metabolic processes, neuropeptide signaling pathways and aminoglycan metabolic processes (Fig. 2C).

The DEGs in FL5W0 vs FL5D5 were enriched in the cellular amide metabolic process, amide biosynthetic process and translation (Fig. 2D). Tissues began to undergo autophagy and apoptosis during the prepupal stage. Ribosomal proteins play a vital role in protein synthesis [48]. The transcription levels of most ribosomal protein genes decreased from the initiation of the wandering stage to the prepupal stage (L5W0 to L5D5) (Additional file 4: Table S4), indicating that protein synthesis reduced during the prepupal stage.

#### The later phase of metamorphosis

Figures 2E and F show the GO enriched categories of the last instar larvae of *H. armigera* from the prepupal stage to the initiation of the pupal stage (L5D5 to P0). At the initiation of the pupal stage, the last instar larvae molted and became pupae (Fig. 1A: P0). GO enrichment analysis revealed that the DEGs in BL5D5 vs BP0 were enriched in chitin metabolic processes, aminoglycan metabolic processes and glucosamine-containing compound metabolic processes (Fig. 2E). The DEGs in FL5D5 vs FP0 were enriched in carbohydrate metabolic processes, oxoacid metabolic processes and carboxylic acid metabolic processes (Fig. 2F).

### Genes related to the JH and ecdysone signaling pathways

**Fig. 3:**
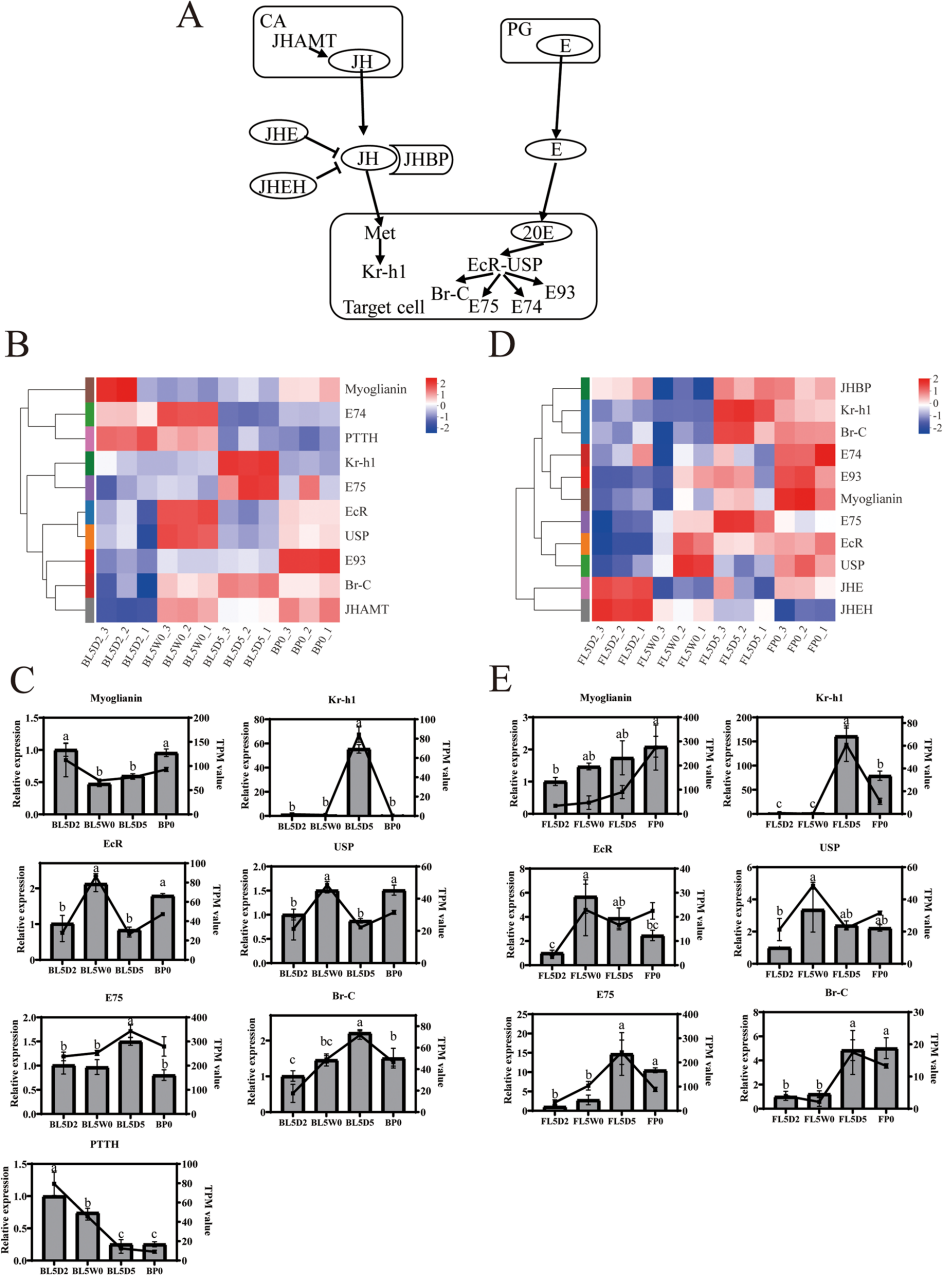
Expression of genes related to the JH and ecdysone signaling pathways during metamorphosis of *Helicoverpa armigera*. **A** The regulatory model of juvenile hormone (JH) and 20E signaling pathways. **B** and **D** Heatmap of genes in the brain (**B**) and fat body (**D**) related to the JH and ecdysone signaling pathways. **C** and **E** qPCR validation of genes in the brain (**C**) and fat body (**E**) related to regulation of JH and ecdysone signaling pathways. Histograms represent the results of qPCR (left Y axis). Line charts represent the results of RNA-Seq (right Y axis). Error bars represent mean ± SD. One-way ANOVA with Tukey’s post hoc test was used for statistical analysis of qPCR results. Different lowercase letters indicated significant differences (*p* < 0.05). BL5D2: the brain at the feeding stage; BL5W0: the brain at the initiation of the wandering stage; BL5D5: the brain at the prepupal stage; BP0: the brain at the initiation of the pupal stage; FL5D2: the fat body at the feeding stage; FL5W0: the fat body at the initiation of the wandering stage; FL5D5: the fat body at the prepupal stage; FP0: the fat body at the initiation of the pupal stage

The JH and ecdysone signaling pathways are the key factors determining insect metamorphosis from larvae to pupae. Based on previous reports [4, 10–13, 49–52], the regulatory model of JH and ecdysone signaling pathways is shown in Fig. 3A. The changes in hormone titers can be indicated by the expression levels of the early JH and ecdysone response genes. The early JH-response gene *Krüppel homolog 1* (*Kr-h1*) in the brain and fat body was highly expressed at the prepupal stage (L5D5) compared with other stages (Figs. 3B, C, D and E; additional file 5: Table S5), implying that JH rose during the prepupal stage. The *JHE* and *JHEH* genes in the fat body were highly expressed at the feeding stage (L5D2) in comparison with the initiation of the wandering stage (L5W0) (Fig. 3D; additional file 5: Table S5), implying that they played a role in decreasing the level of JH during the feeding stage. *JHBP* in the fat body was expressed at lower levels at the initiation of the wandering stage (L5W0) than at other stages (Fig. 3D; additional file 5: Table S5). *Broad-Complex* (*Br–C*) and *ecdysone hormone-response protein 75* (*E75*) were highly expressed at the prepupal stage (L5D5) compared with the initiation of the wandering stage (L5W0) (Figs. 3B, C, D and E; additional file 5: Table S5). The expression level of *PTTH* in the brain was high at the feeding stage (L5D2) and the initiation of the wandering stage (L5W0) and decreased at the prepupal stage (L5D5) and the initiation of the pupal stage (P0) (Figs. 3B and C; additional file 5: Table S5).

### Genes related to neuropeptides and hormone (or neuropeptide) receptors

**Fig. 4:**
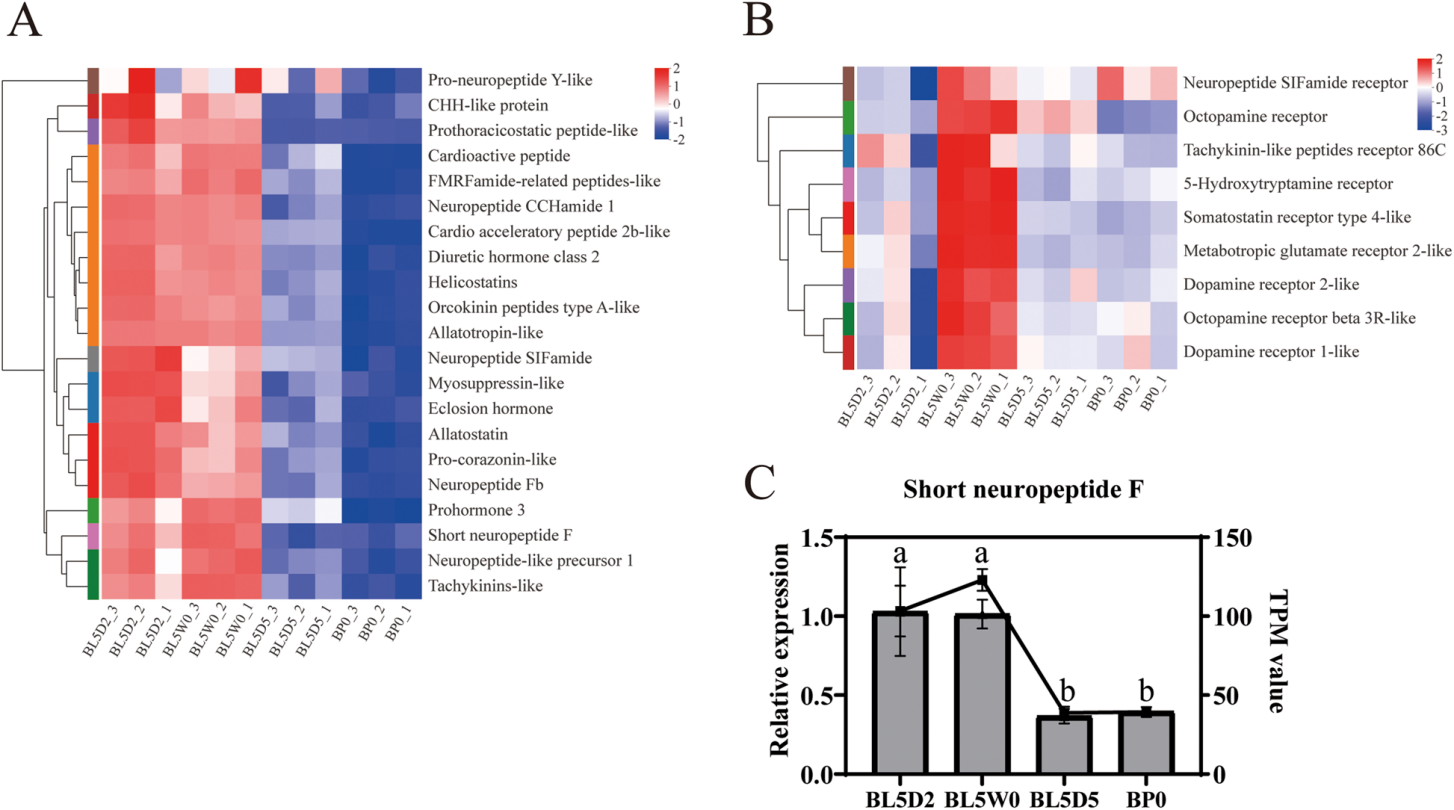
Expression of genes related to neuropeptides and hormone (or neuropeptide) receptors during metamorphosis of *Helicoverpa armigera*. **A** and **B** Heatmap of genes in the brain related to neuropeptides (**A**) and hormone (or neuropeptide) receptors (**B**). **C** qPCR validation of a gene in the brain related to neuropeptide. Histograms represent the results of qPCR (left Y axis). Line charts represent the results of RNA-Seq (right Y axis). Error bars represent mean ± SD. One-way ANOVA with Tukey's post hoc test was used for statistical analysis of qPCR results. Different lowercase letters indicated significant differences (*p* < 0.05). BL5D2: the brain at the feeding stage; BL5W0: the brain at the initiation of the wandering stage; BL5D5: the brain at the prepupal stage; BP0: the brain at the initiation of the pupal stage

Various neuropeptide genes in the brain of *H. armigera* were highly expressed at the feeding stage (L5D2) and the initiation of the wandering stage (L5W0) and less expressed at the prepupal stage (L5D5) and the initiation of the pupal stage (P0) (Fig. 4A and C; additional file 6: Table S5), indicating that these neuropeptides played important roles in the physiological processes during the feeding stage and the initiation of the wandering stage. The transcription levels of many hormone (or neuropeptide) receptors, including receptors of dopamine, octopamine, neuropeptide SIFamide, 5-hydroxytryptamine and somatostatin, were specifically increased at the initiation of the wandering stage (L5W0) in comparison with other stages (Fig. 4B; additional file 6: Table S6). At the initiation of the wandering stage, cotton bollworm larvae usually empty their intestines and actively crawl around to find a place to build a pupal chamber, with which they prepare for the initiation of metamorphosis. The enhanced expression levels of these hormone (or neuropeptide) receptors may be related to the series of sophisticated behaviors at this stage.

### Genes related to autophagy

**Fig. 5:**
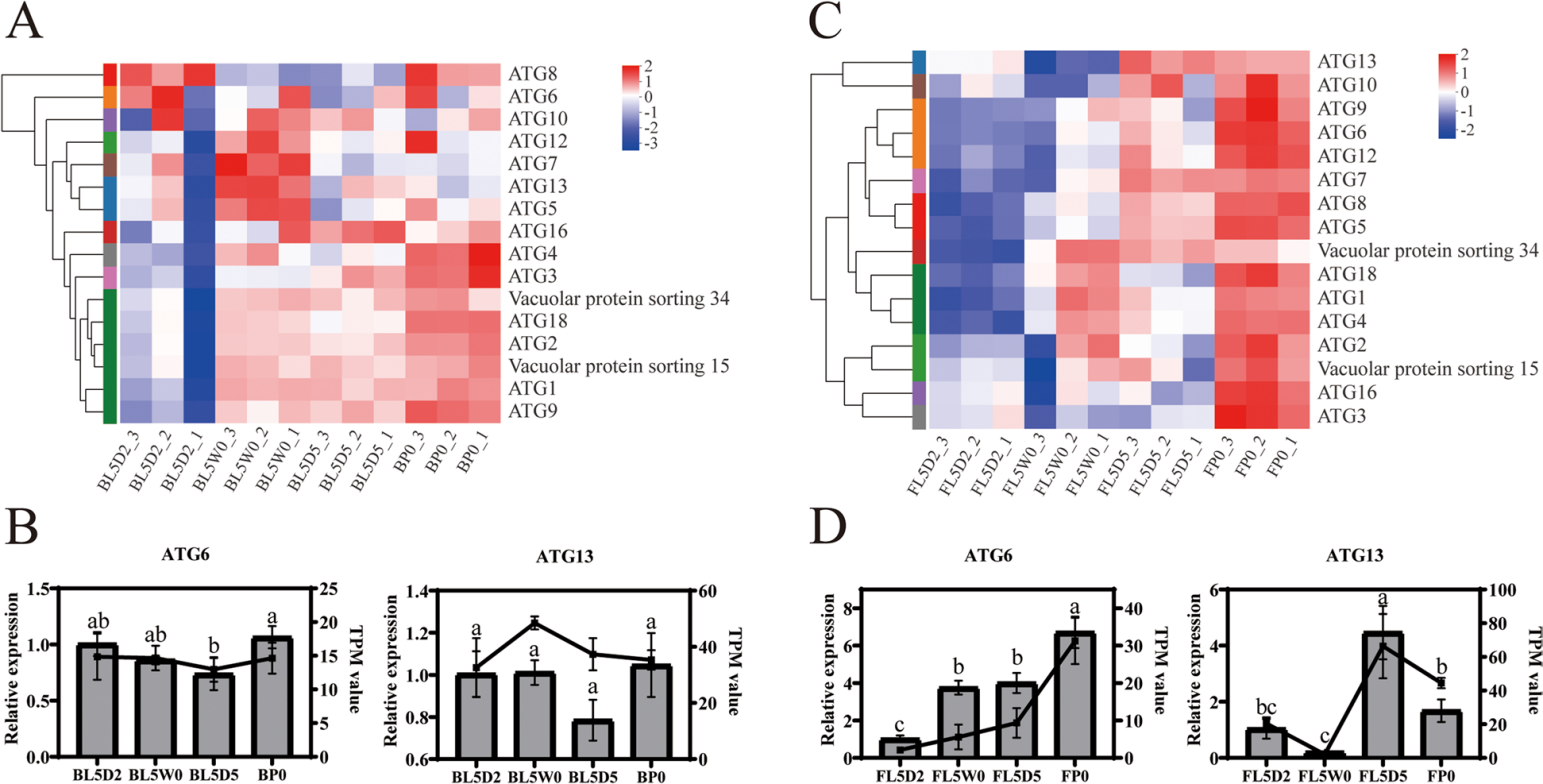
Expression of genes related to autophagy during metamorphosis of *Helicoverpa armigera*. **A** and **C** Heatmap of genes in the brain (**A**) and fat body (**C**) related to autophagy. **B** and **D**: qPCR validation of genes in the brain (**B**) and fat body (**D**) related to autophagy. Histograms represent the results of qPCR (left Y axis). Line charts represent the results of RNA-Seq (right Y axis). Error bars represent mean ± SD. One-way ANOVA with Tukey’s post hoc test was used for statistical analysis of qPCR results. Different lowercase letters indicated significant differences (*p* < 0.05). BL5D2: the brain at the feeding stage; BL5W0: the brain at the initiation of the wandering stage; BL5D5: the brain at the prepupal stage; BP0: the brain at the initiation of the pupal stage; FL5D2: the fat body at the feeding stage; FL5W0: the fat body at the initiation of the wandering stage; FL5D5: the fat body at the prepupal stage; FP0: the fat body at the initiation of the pupal stage

The Atg1-Atg13 complex is involved in the process of autophagosome initiation [53]. In *H. armigera*, 14 ATGs were identified. Although the transcription levels of several ATGs (ATG5, ATG7, ATG12 and ATG13) in the brain at the initiation of the wandering stage (L5W0) were higher in comparison with other stages (Figs. 5A and B; additional file 7: Table S7), there were few differences in the expression levels (less than 1.5-fold change in pairwise comparisons) at different stages. The transcription levels of many ATGs (ATG5, ATG6, ATG7, ATG8, ATG9, ATG12) in the fat body gradually increased from the feeding stage (L5D2) to the initiation of the pupal stage (P0) (Figs. 5C and D; additional file 7: Table S7). The mRNA levels of *ATG1, ATG4,* and *ATG18* in the fat body decreased from the initiation of the wandering stage to the prepupal stage (L5W0 to L5D5) (Fig. 5C; additional file 7: Table S7).

### Genes related to apoptosis and proliferation

**Fig. 6:**
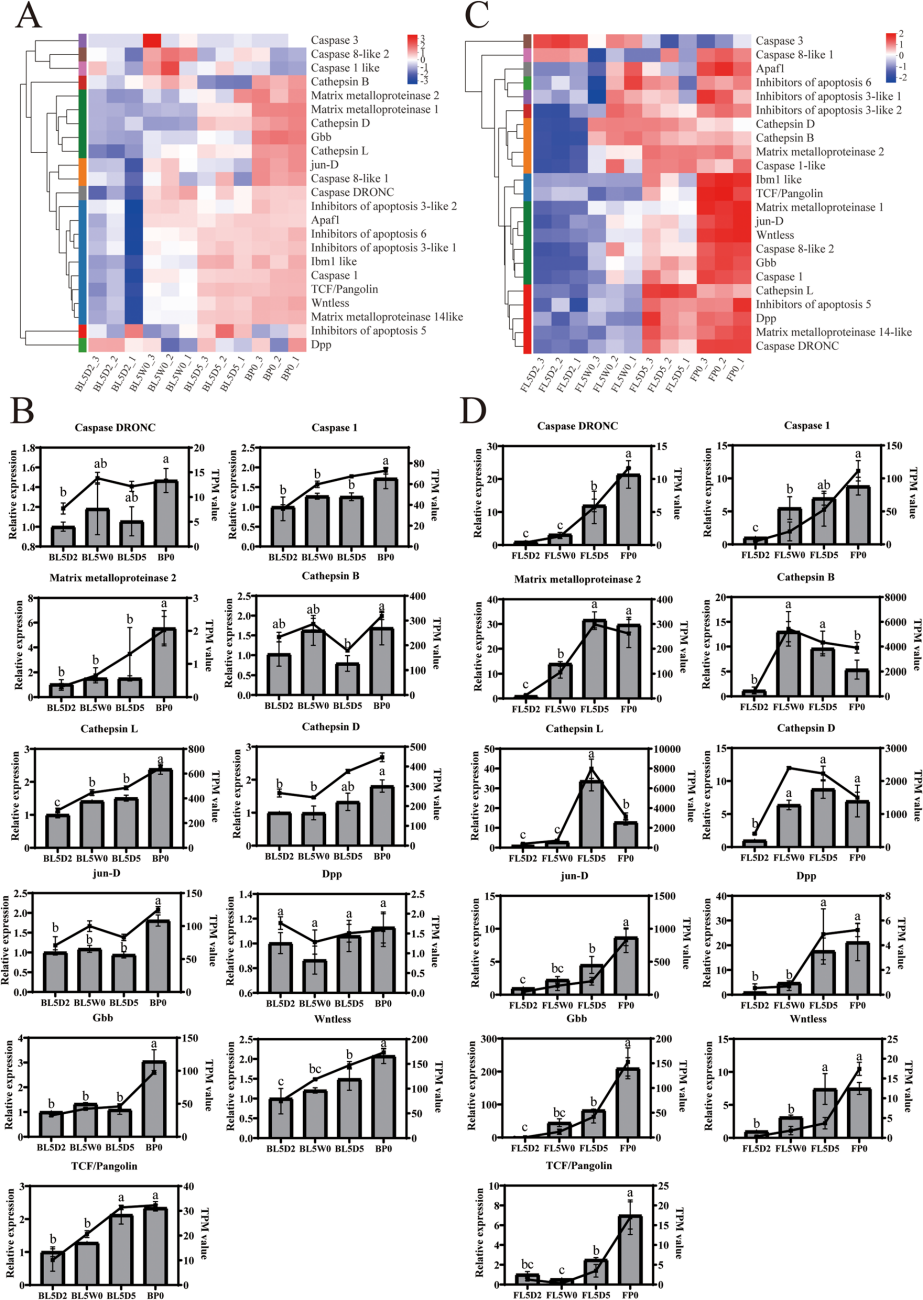
Expression of genes related to apoptosis and proliferation during metamorphosis of *Helicoverpa armigera*. **A** and **C** Heatmap of genes in the brain (**A**) and fat body (**C**) related to apoptosis and proliferation. **B** and **D** qPCR validation of genes in the brain (**B**) and fat body (**D**) related to apoptosis and proliferation. Histograms represent the results of qPCR (left Y axis). Line charts represent the results of RNA-Seq (right Y axis). Error bars represent mean ± SD. One-way ANOVA with Tukey’s post hoc test was used for statistical analysis of qPCR results. Different lowercase letters indicated significant differences (*p* < 0.05). BL5D2: the brain at the feeding stage; BL5W0: the brain at the initiation of the wandering stage; BL5D5: the brain at the prepupal stage; BP0: the brain at the initiation of the pupal stage; FL5D2: the fat body at the feeding stage; FL5W0: the fat body at the initiation of the wandering stage; FL5D5: the fat body at the prepupal stage; FP0: the fat body at the initiation of the pupal stage

The larval tissues are removed through caspase-induced apoptosis, and new pupal-adult tissues are formed via proliferation under the control of E [3]. The transcription levels of *caspase DRONC* and *caspase 1* in the brain and fat body were enhanced from the feeding stage to the initiation of the pupal stage (L5D2 to P0) (Figs. 6A, B, C and D; additional file 8: Table S8), indicating that apoptosis was active in cotton bollworm cells during metamorphosis. Three genes encoding matrix metalloproteinases (matrix metalloproteinase 1, 2 and 14-like) in the brain and fat body were upregulated during metamorphosis (Figs. 6A and C; additional file 8: Table S8). *Cathepsin B*, *D*, and *L* genes in the fat body were highly expressed during metamorphosis (Figs. 6C and D; additional file 8: Table S8). Interestingly, the transcription level of *cathepsin B* in the brain decreased from the initiation of the wandering stage to the prepupal stage (L5W0 to L5D5) (Figs. 6A and B; additional file 8: Table S8).

**Fig. 7:**
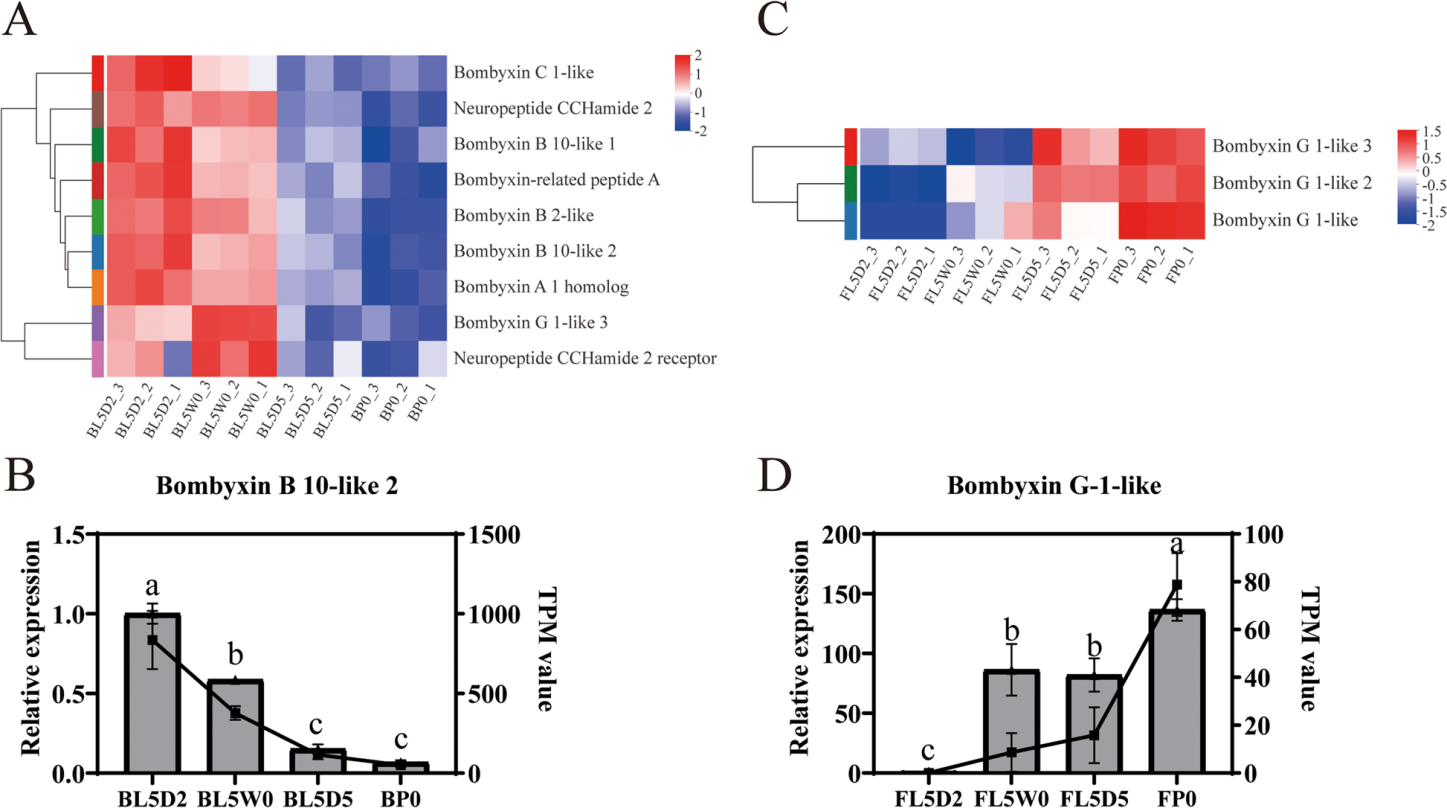
Expression of genes related to the insulin-signaling pathway during metamorphosis of *Helicoverpa armigera*. **A** and **C** Heatmap of genes in the brain (**A**) and fat body (**C**) related to the insulin-signaling pathway. **B** and **D** qPCR validation of genes in the brain (**B**) and fat body (**D**) related to the insulin-signaling pathway. Histograms represent the results of qPCR (left Y axis). Line charts represent the results of RNA-Seq (right Y axis). Error bars represent mean ± SD. One-way ANOVA with Tukey’s post hoc test was used for statistical analysis of qPCR results. Different lowercase letters indicated significant differences (*p* < 0.05). BL5D2: the brain at the feeding stage; BL5W0: the brain at the initiation of the wandering stage; BL5D5: the brain at the prepupal stage; BP0: the brain at the initiation of the pupal stage; FL5D2: the fat body at the feeding stage; FL5W0: the fat body at the initiation of the wandering stage; FL5D5: the fat body at the prepupal stage; FP0: the fat body at the initiation of the pupal stage

JAK/STAT [54], TGF-β [55], Wnt/β-catenin [56], and insulin signaling [57] are involved in the differentiation and proliferation of stem cells. Many genes in *H. armigera* related to cell differentiation and proliferation were highly expressed at the initiation of the pupal stage (P0), including *jun-D*, *Dpp*, *glass bottom boat* (*Gbb*), *wntless*, *TCF/Pangolin* and insulin-like peptides from the fat body (Figs. 6A, B, C and D, 7C and D; additional file 8: Table S8; additional file 9: Table S9).

### Insulin-signaling pathway

A total of nine insulin-like peptides (bombyxin C 1-like, B 10-like 1, B 10-like 2, A 1 homolog, B 2-like, related peptide A, G 1-like, G 1-like 2 and G 1-like 3) were identified from our transcriptome data (additional file 9: Table S9). Six insulin-like peptides (bombyxin C 1-like, B 10-like 1, B 10-like 2, A 1 homolog, B 2-like and related peptide A) were specifically expressed in the brain (Figs. 7A and B; additional file 9: Table S9), and two insulin-like peptides (bombyxin G 1-like and bombyxin G 1-like 2 [58]) were specifically expressed in the fat body (Figs. 7C and D; additional file 9: Table S9). One insulin-like peptide (bombyxin G 1-like 3 [58]) was expressed in both the brain and the fat body (Figs. 7A and C; additional file 9: Table S9). Insulin-like peptides from the brain were strongly expressed at the feeding stage (L5D2) and the initiation of the wandering stage (L5W0) in comparison with other stages (Figs. 7A and B; additional file 9: Table S9). During insect feeding, the intake of sugars and amino acids facilitates the secretion of insulin-like peptides from the brain by promoting the secretion of CCHamide 2 in the fat body [59]. The expression of *CCHamide 2* in the brain (Fig. 7A; additional file 9: Table S9), rather than in the fat body, was consistent with the expression pattern of insulin-like peptides in the brain. The expression of insulin-like peptides from the brain at the initiation of the wandering stage (L5W0) may be associated with the secretion of E. Three insulin-like peptides (bombyxin G 1-like, G 1-like 2 and G 1-like 3) in the fat body highly expressed at the initiation of the pupal stage (Figs. 7C and D; additional file 9: Table S9), indicating that insulin-like peptides from the fat body were probably associated with active cell proliferation during the metamorphosis of *H. armigera*.

## Discussion

### Changes in the expression genes and signaling pathways related to hormone signaling

According to the change in JH titer in lepidopteran insect from larva to pupa [2] and the transcription level change of *Kr-h1* (Figs. 3B, C, D and E; additional file 5: Table S5), the level of JH in the last instar larvae of cotton bollworm was predicted to maintain at a low level at the feeding stage and the initiation of the wandering stage, rise to a high level during the prepupal stage, and decrease to a low level at the initiation of the pupal stage. The titer of JH in the last instar of lepidopteran larvae is related to the transcription of *JHAMT* [4]. In *Bombyx mori*, the transcription level of *JHAMT* decreases rapidly and disappears in CA of the last instar larvae. However, other peripheral tissues still have JHAMT activity which is probably the reason causing a high level of JH in the larval hemolymph during the prepupal stage [4]. It is too hard to collect enough CA, thus we only detected *JHAMT* expression in the brain without corpus cardiacum and CA. The expression level of JHAMT in the CA of *H. armigera* was not clear in this study. Whether the expression level of JHAMT in the brain (no corpus cardiacum and CA) (Fig. 3B; additional file 5: Table S5) was related to JH titer needs to be further studied. Myoglianin regulates JHAMT transcription in CA of *Gryllus bimaculatus* to control JH levels to trigger metamorphosis [6]. Myoglianin is considered to triggered metamorphosis at the threshold size in *Manduca sexta* and *T. castaneum* [7]. The function of myoglianin in *H. armigera* needs to be investigated.

**Fig. 8:**
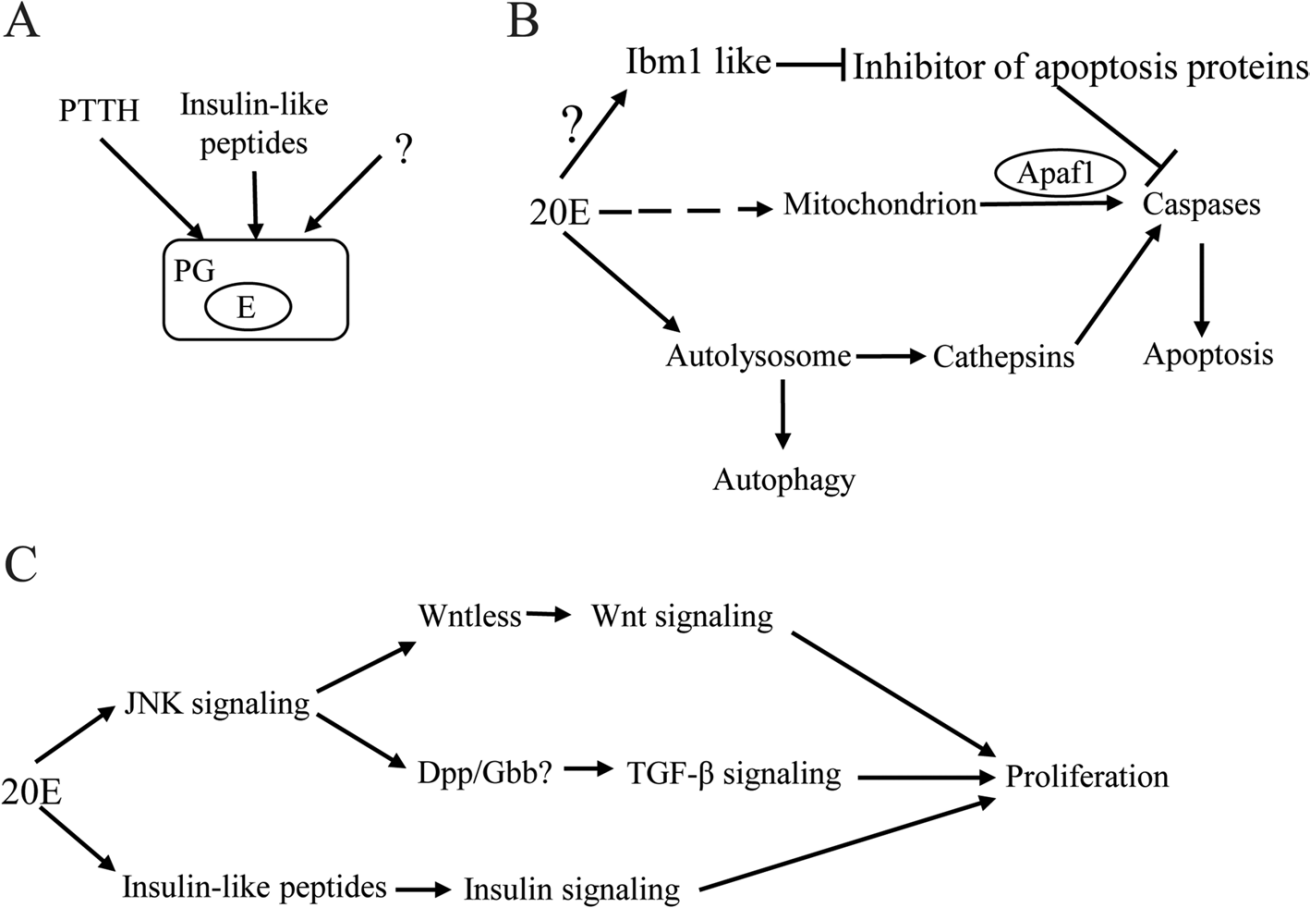
Putative physiological processes of *Helicoverpa armigera* during its metamorphosis. **A** The regulation of ecdysone production. **B** Autophagy and apoptosis induced by 20E. **C** Proliferation induced by 20E. The solid arrow represents direct effect. The dotted arrow represents indirect effect

The secretion of E mainly starts at the initiation of the wandering stage according to previous studies [60]. In *Drosophila*, many factors control the production of E, including PTTH, insulin signaling, TGF-β/Activin signaling, nitric oxide, and prothoracicostatic factors [61]. PTTH and insulin signaling are involved in the production of E in *B. mori*, while insulin signaling is not required for the ecdysone production in *M. sexta* [19]. The transcription levels of *PTTH* (Figs. 3B and C; additional file 5: Table S5) and insulin-like peptides from the brain, except for *bombyxin G1-like 3* (Figs. 7A and B; additional file 9: Table S9), were decreased from the feeding stage to the initiation of the wandering stage (L5D2 to L5W0), indicating that there may be other factors contributing to the biosynthesis of E in the PGs of *H. armigera* (Fig. 8A). JH increases again after the beginning of metamorphosis to prevent excessive metamorphosis by antagonizing 20E [50]. *Kr-h1*, a key JH response gene, may play an important role in preventing excessive metamorphosis in *H. armigera* considering its high expression level at the prepupal stage (Figs. 3B, C, D and E; additional file 5: Table S5). Overexpression of *Kr-h1* reduced the transcription levels of *EcR*, *USP* and other ecdysone response genes in *Drosophila* [22]. The titer of 20E rises from the initiation of the wandering stage to the prepupal stage [60], while the expression levels of the ecdysone receptor *EcR*, *USP* and the *ecdysone response protein 74* (E74) in the brain did not increase from the initiation of the wandering stage to the prepupal stage (L5W0 to L5D5) (Figs. 3B and C; additional file 5: Table S5), indicating that JH antagonizes ecdysone signaling by reducing the transcription of *EcR*, *USP* and *E74* via Kr-h1. On the whole, 20E signaling was activated during the metamorphosis of *H. armigera*. JH signaling was also activated to partially block 20E signaling during the prepupal stage of *H. armigera*.

### Autophagy and apoptosis

When JH is below a certain limit, the secretion of E will cause autophagy and apoptosis. Generally, autophagy and apoptosis signaling were activated from the initiation of the wandering stage to the initiation of the pupal stage, implying an important role of these two signaling pathways in the metamorphosis of *H. armigera*.

Autophagy is a self-protection function of cells under external pressure. Ecdysone response genes induce the expression of autophagy response genes and block PI3K-TORC1 signaling to induce autophagy [53, 62]. The high transcriptional level of *Br–C* and *E75* at the prepupal stage (L5D5) (Figs. 3B, C, D and E; additional file 5: Table S5) implied these two ecdysone response genes may play an important role in inducing autophagy and apoptosis in *H. armigera*. The level of 20E was low at the initiation of the pupal stage according to a previous study [60]. E93, a 20E response gene, upregulates the transcription levels of ATGs in *Drosophila* [34]. E93 inhibited by Kr-h1 and induced by 20E promotes autophagy in the fat body of *B. mori* [63, 64]. The *E93* gene (Figs. 3B and D; additional file 5: Table S5) and many ATGs (Figs. 5A and C; additional file 7: Table S7) in the brain and fat body were highly expressed at the initiation of the pupal stage (P0), suggesting that upregulation of *E93* was related to the induction of ATGs at the initiation of the pupal stage.

20E activates reaper and hid to induce cell apoptosis during *Drosophila* metamorphosis [65]. Ibm1, the reaper/grim ortholog, has been identified in *B. mori* and is associated with cell apoptosis [66]. The transcription of *ibm1-like*, the ibm1 ortholog of *H. armigera*, increased in the brain and fat body from the feeding stage to the initiation of the pupal stage (L5D2 to P0) (Figs. 6A and C; additional file 8: Table S8), indicating that ibm1-like was involved in the activation of apoptosis. Cathepsins are released from the lysosomes into the cytoplasm when stimulated by signals [67]. Lysosomes are involved in the process of autophagy by forming autolysosomes [24]. In addition, autophagy precedes apoptosis, and inhibition of autophagy prevents apoptosis [60]. Cathepsin L and D promote fat body degradation and programmed cell death during the metamorphosis of *B. mori* [68, 69]. Cathepsin L upregulated by 20E participates in the regulation of granulation of haemocytes and triggers apoptosis of midgut cells by activating caspase-1 in *H. armigera* [33, 70]. Mature cathepsin D triggered by autophagy promotes apoptosis via activating caspase-3 in the midgut of *H. armigera* [60]. Considering their high expression levels (Figs. 6C and D; additional file 8: Table S8), *cathepsin B, D,* and *L* in the fat body may play a vital role in the activation of apoptosis in *H. armigera*. These results implied that 20E might induce apoptosis by releasing cathepsins, which were activated by autolysosomes, to activate caspases in *H. armigera*. Furthermore, mitochondria are involved in the apoptosis of lepidopteran insect cells [26, 71–73]. However, there is no evidence that mitochondria are involved in the activation of apoptosis in *Drosophila* [27]. Apaf1 is essential for mitochondria-dependent apoptosis [74], and its expression level in the brain increased during metamorphosis (Fig. 6A; additional file 8: Table S8), indicating that Apaf1 is involved in the activation of apoptosis in cotton bollworm. In conclusion, many factors, including ibm1-like, autolysosome, cathepsins, mitochondria, and Apaf1, are associated with cell apoptosis of *H. armigera* during metamorphosis (Fig. 8B).

### Cell proliferation

Cell proliferation has a crucial role in insect metamorphosis. From larvae to pupae, larval tissues undergo autophagy, apoptosis and cell proliferation under the regulation of the E. Autophagy and apoptosis are essential for the differentiation and proliferation of tissue and stem cells, which act in an ecdysone concentration-dependent manner [75, 76]. The dissociation of fat body cells is involved in the proliferation of fat bodies, which is regulated by 20E through mediating the expression of matrix metalloproteinases and cathepsins [3, 77, 78]. 20E is required for fat body cell dissociation induced by two matrix metalloproteinases in *Drosophila* [78]. Cathepsin L regulated by 20E is involved in the dissociation of fat body cell in *H. armigera* [79]. According to our data (Figs. 6C and D; additional file 8: Table S8), three matrix metalloproteinases (matrix metalloproteinase 1, 2 and 14-like) and three cathepsins (cathepsin B, D and L) in the fat body may play an important role in the dissociation of fat body cells of *H. armigera*. Apoptotic cells need to leave space for the proliferation of stem cells. The Dpp and Wnt signaling pathways play key roles in the differentiation and proliferation of stem cells [80–82]. Wingless promotes cell survival but restricts growth, which determines the final wing size during wing development in *Drosophila* [83]. Inhibition of caspase on apoptotic cells can promote the secretion of Dpp and wingless. Dpp can induce extra proliferation of cells, and wingless can inhibit the extra proliferation caused by Dpp in *Drosophila* [84]. The transcription factor *TCF/Pangolin* in the brain and fat body was highly expressed at the initiation of the pupal stage (P0) (Figs. 6A, B, C and D; additional file 8: Table S8), indicating that Wnt signaling was involved in cell proliferation. Gbb, a BMP ligand, plays an important role in the growth and patterning of *Drosophila* wings [85, 86]. The transcription level of *Dpp* in the fat body was enhanced from the initiation of the wandering stage to the initiation of the pupal stage (L5W0 to P0) (Figs. 6C and D; additional file 8: Table S8), and the transcription level of *Gbb* in the brain and fat body increased from the feeding stage to the initiation of the pupal stage (L5D2 to P0) (Figs. 6A, B, C and D; additional file 8: Table S8), implying that Dpp and Gbb were required for cell proliferation. Activation of the JNK signaling pathway promotes the expression of Dpp and wingless in *Drosophila* [87, 88]. The transcription level of *jun-D* in the brain and fat body increased from the initiation of the wandering stage to the initiation of the pupal stage (L5W0 to P0) (Figs. 6A, B, C and D; additional file 8: Table S8), implying that the JNK pathway is located upstream of Dpp and wingless signaling to activate cell proliferation. 20E activates JNK signaling to control oviposition in *Anopheles gambiae* [89]. In addition, 20E regulates gene expression through an interaction between EcR and Jun during *Drosophila* dorsal closure [90]. As an insulin-like peptide produced by the fat body, insulin-like peptide 6 induced by 20E signaling, starvation and FOXO regulates insect growth and metabolism [3, 91]. Upregulation of the three insulin-like peptides (*bombyxin G1-like*, *G1-like 2* and *G1-like 3*) during metamorphosis in the fat body (Figs. 7C and D; additional file 9: Table S9) indicated their roles in cell proliferation of *H. armigera*. In conclusion, four pathways, including the insulin pathway, JNK pathway, TGF-β pathway, and Wnt pathway, cooperated under the regulation of 20E to promote cell proliferation during *H. armigera* metamorphosis from larvae to pupae (Fig. 8C).

## Conclusions

We mainly studied different processes of metamorphosis by conducting a comprehensive analysis of the transcriptome at different stages in the last larval instar of *H. armigera*. Transcriptome analysis revealed specific gene expression patterns at different stages of metamorphosis. The expression levels of neuropeptides were downregulated during metamorphosis. Our study showed the specific process of the regulation of JH and ecdysone signaling pathways and the changes in genes related to autophagy at different stages. In addition, our study showed the levels of genes and signaling pathways related to apoptosis and cell proliferation. The molecular mechanisms of cell apoptosis and proliferation during insect metamorphosis from larvae to pupae need to be identified in the future.

## Materials and methods

### Insect rearing and tissue collection

*H. armigera* were purchased from Henan Jiyuan Baiyun Industry in China. *H. armigera* larvae were raised on an artificial diet and a photoperiod of 14 h light:10 h dark at a temperature of 27 ± 1 °C with 60% to 70% humidity. Three biological replicates of the brain (no corpus cardiacum and CA) (each biological replicate included 50 individuals) and the fat body (each biological replicate including 10 individuals) were collected at L5D2 (the feeding stage; the second day of the fifth instar larvae), L5W0 (the initiation of the wandering stage; the third day of the fifth instar larvae), L5D5 (the prepupal stage; the fifth day of the fifth instar larvae), and P0 (the initiation of the pupal stage) in the last instar of *H. armigera* larvae.

### RNA extraction and library construction and sequencing

Total RNA of the brain and fat body tissues was extracted using TRIzol reagent (Invitrogen, USA) according to the manufacturer’s instructions. The RNA quality was determined using a Nanodrop ND-2000 spectrophotometer, and the RNA integrity was evaluated using an Agilent 2100 Bioanalyzer (Agilent, Santa Clara, CA, USA). A cDNA library was constructed using 1 μg of RNA following the Truseq™ RNA Sample Preparation Kit (Illumina, San Diego, CA, USA). Finally, the constructed cDNA libraries were sequenced on an Illumina HiSeq xten/NovaSeq 6000 sequencer (Majorbio, Shanghai, China).

### Data analysis

Low-quality and adaptor-polluted reads and high levels of unknown base (N) reads were removed to obtain clean reads. The clean reads were aligned to the *H. armigera* reference genome (GCF_002156985.1) with orientation mode using HISAT2 [92]. StringTie [93] was used to assemble transcripts, and novel transcripts were identified by Cuffcompare [94]. This assembly contains 19,157 predicted protein-coding genes. The expression levels of genes were calculated by transcripts per million reads (TPM). Differentially expressed genes (DEGs) in the 24 samples were identified using the DESeq2 R package [95]. DEGs with |Log2 (fold change)|≥ 1 and FDR adjusted *P value* < 0.05 were identified as significant DEGs. Gene Ontology (GO) analysis was performed to assess possible gene functions [96]. DEGs were enriched in three GO groups to produce a summary of the gene groups within the transcriptome. GO terms with the FDR adjusted *P value* < 0.05 were considered significantly enriched. Average linkage hierarchical clustering analyses were conducted to visualize the expression profiles of metamorphosis-related genes using the RSEM software.

### Quantitative reverse transcription PCR (RT–qPCR)

A total of 1.2 μg of RNA was used for cDNA synthesis using the SuperRT cDNA Kit (CWBIO, China). Quantitative real-time PCR (qPCR) was performed on a QuantStudio 12 K Flex Real-time PCR System (Applied Biosystems, USA). The ribosomal protein S3 (rpS3) gene was used as the reference gene [97]. The qPCR conditions were 95 °C for 30 s, followed by 40 cycles of 95 °C for 5 s and 60 °C for 30 s. Three biological replicates were performed for each treatment, and each biological replicate was carried out in triplicate. The expression levels of genes were calculated using the 2^−ΔΔct^ method, and One-way ANOVA with Tukey's post hoc test was used for statistical analysis. The primers used in the qPCR are described in Additional file 10: Table S10.

## Supplementary Information

**Additional file 1:** **Table S1.** All samples for the transcriptome of cotton bollworm at different stages using RNA-seq.

**Additional file 2:** **Table S2.** Summary of clean reads mapped to the reference *H. armigera* genome.

**Additional file 3: Table S3.** All DEGs of *H. armigera* at different stages.

**Additional file 4:** **Table S4.** DEGs (BL5D2_vs_BL5W0; FL5D2_vs_FL5W0; FL5W0_vs_FL5D5) identified by GO enrichment analysis.

**Additional file 5:** **Table S5.** Genes related to regulation of juvenile hormone and ecdysone signaling pathways.

**Additional file 6:** **Table S6.** Genes related to neuropeptides and hormone (or neuropeptide) receptors.

**Additional file 7:** **Table S7.** Genes related to autophagy.

**Additional file 8:** **Table S8.** Genes related to apoptosis and proliferation.

**Additional file 9:** **Table S9.** Genes related to the insulin-signaling pathway.

**Additional file 10:** **Table S10.** All primers for qPCR.

## Data Availability

The *H. armigera* raw sequencing data were deposited in the Genome Sequence Archive in National Genomics Data Center, Beijing Institute of Genomics, Chinese Academy of Sciences (GSA: CRA006668) that are publicly accessible at https://ngdc.cncb.ac.cn/gsa.

## Abbreviations

DEGs: Differentially expressed genes
20E: 20-Hydroxyecdysone
JH: Juvenile hormone
ATGs: Autophagy-related genes
JHAMT: Juvenile hormone acid methyltransferase
Dpp: Decapentaplegic
Gbb: Glass bottom boat
Kr-h1: Krüppel homolog 1
JHE: Juvenile hormone esterase
JHEH: Juvenile hormone epoxide hydrolase
JHBP: Juvenile hormone binding protein
PTTH: Prothoracicotropic hormone
EcR: Ecdysone receptor
USP: Ultraspiracle
Br–C: Broad-complex
E74: Ecdysone-induced protein 74
E75: Ecdysone-induced protein 75
E93: Ecdysone-induced protein 93
IAPs: Inhibitors of apoptosis
Ibm1 like: IAP-binding motif 1 like
rpS3: Ribosomal protein S3
CA: Corpora allata
E: Ecdysone
PG: Prothoracic gland
Met: Methoprene-tolerant
